# Multidrug-Resistant *Salmonella* Serotype Anatum in Travelers and Seafood from Asia, United States

**DOI:** 10.3201/eid2605.190992

**Published:** 2020-05

**Authors:** Beth E. Karp, Molly M. Leeper, Jessica C. Chen, Kaitlin A. Tagg, Louise K. Francois Watkins, Cindy R. Friedman

**Affiliations:** Centers for Disease Control and Prevention, Atlanta, Georgia, USA (B.E. Karp, M.M. Leeper, J.C. Chen, L.K.Francois Watkins, C.R. Friedman);; Weems Design Studio, Inc., Suwanee, Georgia, USA (K.A. Tagg)

**Keywords:** *Salmonella*, Anatum, antimicrobial resistance, MDR, bacteria, travel, imported, foodborne diseases, food safety, seafood, DHA-1, *bla*_DHA-1_, beta-lactamases, tilapia, shrimp, Asia, United States, Salmonella enterica serotype Anatum

## Abstract

A multidrug-resistant *Salmonella enterica* serotype Anatum strain reported in Taiwan was isolated in the United States from patients and from seafood imported from Asia. Isolates harbored 11 resistance determinants, including quinolone and inducible cephalosporin resistance genes. Most patients had traveled to Asia. These findings underscore the need for global One Health resistance surveillance.

A sharp increase in *Salmonella enterica* serotype Anatum infections reported in Taiwan during 2016–2017 was associated with emergence of multidrug-resistant (MDR) strains harboring 11 resistance genes: *aadA2*, *bla*_DHA-1_, *dfrA23*, *floR*, *lnu*(F), *qnrB4*, *strA*, *strB*, *sul1*, *sul2*, and *tet*(A) (*1*). Isolates had intermediate susceptibility to ciprofloxacin and resistance to many antimicrobial agents, including third-generation cephalosporins. We report human cases and related isolates in the United States.

We found 43 isolates genetically related to MDR *Salmonella* Anatum from Taiwan in the National Center for Biotechnology Information Pathogen Detection Isolates Browser (http://www.ncbi.nlm.nih.gov/pathogens). We analyzed genome assemblies for resistance determinants and plasmids by using databases adapted from ResFinder and PlasmidFinder (Center for Genomic Epidemiology, https://cge.cbs.dtu.dk). To assess strain relatedness, we constructed a core genome multilocus sequence typing (cgMLST) phylogenetic tree and pairwise matrix of allele differences by using BioNumerics version 7.6 (Applied Maths, http://www.applied-maths.com). We contacted US health departments to obtain patient information and isolates for susceptibility testing by broth microdilution (Appendix Table 1).

We created a cgMLST phylogenetic tree showing resistance determinants detected for 40 isolates with >99.5% similarity and 0–20 allele differences (Figure; Appendix Figure). We excluded 3 more distantly related isolates. A total of 25 isolates were from Taiwan (16 from humans, 3 each from chickens and pigs, 2 from geese, and 1 from a duck); 12 were from the United States (7 from humans, 4 from tilapia imported from Taiwan, and 1 from shrimp imported from the Philippines). We detected IncC plasmids in all isolates, except PNUSAS038936; 15 had additional plasmids (Appendix Table 2). Most (38/40) had the previously reported 11 resistance genes (*1*). Two isolates from the Philippines had additional resistance genes, including *mph*(A), *qnrA6*, and *oqxAB*; 3 isolates from tilapia in the United States and 1 human isolate from Taiwan had *mcr-1.1*. We found no quinolone resistance–determining region mutations.

**Figure:**
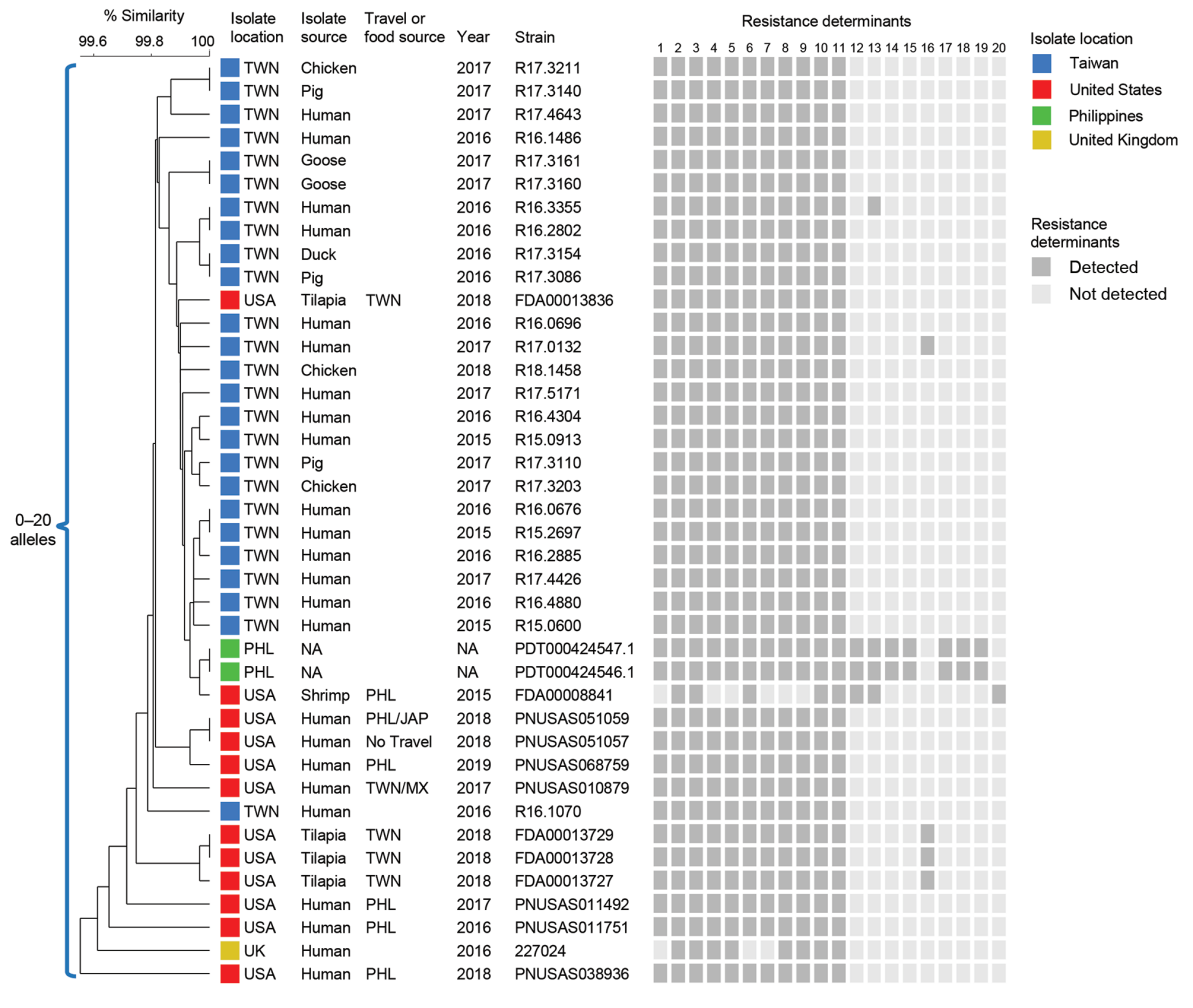
Core genome multilocus sequence typing (cgMLST) phylogenetic tree of 40 *Salmonella enterica* serotype Anatum isolates, 2015–2019. The tree was constructed by using BioNumerics version 7.6 (Applied Maths, http://www.applied-maths.com). Isolate sources, collection years, and National Center for Biotechnology Information strain or isolate numbers are shown. For isolates from the United States, international travel destinations of patients and sources of imported foods are provided. Dark gray boxes indicate resistance determinants detected: 1) *aadA2*; 2) *aph(3″)-Ib* (*strA*); 3) *aph (**6**)-Id* (*strB*); 4) *bla*_DHA-1_; 5) *dfrA23*; 6) *floR*; 7) *lnu*(F); 8) *qnrB4*; 9) *sul1*; 10) *sul2*; 11) *tet*(A); 12) *aadA1*; 13) *bla*_TEM-1B_; 14) *dfrA1*; 15) *dfrA12*; 16) *mcr-1.1*; 17) *mph*(A); 18) *oqxAB*; 19) *qnrA6*; 20) *sul3*. Scale bar indicates percentage similarity. JAP, Japan; MX, Mexico; NA, not available; PHL, Philippines; TWN, Taiwan; UK, United Kingdom; USA, United States.

The 7 patients from the United States were 19–71 (median 48) years of age; 3 were women and 4 men. Among 5 patients with data on race, 3 were Asian and 2 white. All patients reported illness, including diarrhea (7/7), abdominal pain (4/7), nausea (2/7), and fever (1/7). None were hospitalized or died. Four became ill <3 days after returning from travel to the Philippines; 1 visited Japan before the Philippines. Two additional patients reported travel before illness onset; 1 traveled to the Philippines and the other to Taiwan and Mexico, but travel and illness onset dates were unavailable. 

One patient had never travelled internationally. Her isolate was indistinguishable from 1 from a patient who traveled to Asia and differed by only 2 alleles from an isolate from shrimp imported from the Philippines. Before illness onset, she ate at several restaurants and had shrimp at an Asian restaurant and sushi bar.

In patient isolates from the United States, *bla*_DHA-1_ appeared to be carried in a complex integron, with the regulatory *ampR* gene positioned upstream and *qnrB4* downstream. Six isolates had IncC plasmids similar to pR16.0676_90k (GenBank accession no. CP029802) (*1*), which likely carried all 11 resistance genes, but long-read sequencing is required for confirmation. Isolate PNUSAS038936 lacked the IncC plasmid replicon but appeared to have an IS*26*-mediated integration of the entire resistance region from the plasmid (≈60 kb) into the chromosome. 

We performed antimicrobial susceptibility testing on 6 patient isolates, including PNUSAS038936. All had intermediate susceptibility to ciprofloxacin (MIC 0.25 µg/mL) and were resistant to amoxicillin/clavulanic acid, ampicillin, cefoxitin, chloramphenicol, streptomycin, sulfisoxazole, tetracycline, and trimethoprim/sulfamethoxazole. One isolate had intermediate susceptibility to ceftriaxone (MIC 2 µg/mL) and 5 were ceftriaxone susceptible; 1 had a MIC of <0.25 µg/mL, 3 MICs of 0.5 µg/mL, and 1 a MIC of 1 µg/mL.

The emergence and spread of *Salmonella* carrying *bla*_DHA-1_ has both clinical and public health implications. Unlike most plasmid-mediated AmpC β-lactamase genes, *bla*_DHA-1_ is inducible (*2*,*3*), which can complicate detection and treatment. Isolates can appear susceptible to third-generation cephalosporins in vitro, but treatment may fail if AmpC induction occurs (*3*,*4*). The co-occurrence of *bla*_DHA-1_; the plasmid-mediated quinolone resistance gene *qnrB4*; and in the isolates from the Philippines, *mph*(A), a macrolide-resistance gene, is worrisome because third-generation cephalosporins (e.g., ceftriaxone), fluoroquinolones (e.g., ciprofloxacin), and the macrolide azithromycin are recommended for *Salmonella* infections requiring treatment (*5*,*6*). In addition, the presence of *mcr-1.1*, which confers resistance to colistin, a drug of last resort for treating MDR gram-negative bacterial infections, is concerning.

Our findings underscore the need for global, One Health surveillance. Most infections likely were acquired during travel in Asia. International travel, particularly to Asia, has been associated with acquisition of *Salmonella* with clinically important resistance (*7*,*8*). Resistance also can be disseminated via food and animals. Imported food likely was the source of infection for 1 patient without international travel. Among imported foods tested by the US Food and Drug Administration, seafood from Asia is a frequently reported source of antimicrobial-resistant *Salmonella* (*9*,*10*). Given the extent of international travel and trade, data sharing among human health, animal health, and food production sectors and across geographic borders is essential to detect MDR strains and inform strategies and interventions to prevent spread.

Appendix Additional information on MDR *Salmonella* Anatum in travelers and seafood imported from Asia to the United States.
