# Supplementary material for: Multidrug-Resistant Salmonella Serotype Anatum in Travelers and Seafood from Asia, United States
Source: Emerg Infect Dis. 2020 May;26(5):1030–3. doi: 10.3201/eid2605.190992 (PMC7181910; doi:10.3201/eid2605.190992)
Supplement: Appendix — Additional information on MDR Salmonella Anatum in travelers and seafood imported from Asia to the United States. [file 19-0992-Techapp-s1.pdf]

Article DOI: https://doi.org/10.3201/eid2605.190992

Multidrug Resistant *Salmonella* Serotype Anatum in Travelers and Seafood from Asia, United States

Appendix

Appendix Table 1. Antimicrobial agents, concentration ranges, and breakpoints used for susceptibility testing of *Salmonella* Anatum isolates, United States, 2016–2019*

CLSI antimicrobial class	Antimicrobial agent	Concentration range, µg/mL	Interpretive categories and MIC breakpoints, µg/mL		
			Susceptible	Intermediate	Resistant
Aminoglycosides	Gentamicin	0.25–16	<4	8	>16
	Streptomycin†	2–64	<16	NA	>32
β-lactam combination agents	Amoxicillin-clavulanic acid	1/0.5–32/16	<8/4	16/8	>32/16
Cephems	Cefoxitin	0.5–32	<8	16	>32
	Ceftriaxone	0.25–64	<1	2	>4
Folate pathway antagonists	Sulfisoxazole	16–256	<256	NA	>512
	Trimethoprim-sulfamethoxazole	0.12/2.38–4/76	<2/38	NA	>4/76
Macrolides	Azithromycin†	0.25–32	<16	NA	>32
Penems	Meropenem	0.06–4	<1	2	>4
Penicillins	Ampicillin	1–32	<8	16	>32
Phenicols	Chloramphenicol	2–32	<8	16	>32
Quinolones	Ciprofloxacin	0.015–4	<0.06	0.12–0.5	>1
	Nalidixic acid	0.5–32	<16	NA	>32
Tetracycline	Tetracycline	4–32	<4	8	>16

*CLSI, Clinical and Laboratory Standards Institute; NA, not applicable (no intermediate MIC range exists)
†CLSI breakpoints are not established for azithromycin and streptomycin; interpretive standards used are breakpoints established by the National Antimicrobial Resistance Monitoring System for resistance monitoring and should not be used to predict clinical efficacy.

Appendix Table 2. Plasmid replicons and resistance determinants detected among 40 *Salmonella* Anatum isolates

Strain	Plasmid replicons	Resistance determinants
227024	IncC	*aph(3'')-Ib aph(6)-Id bla*$_{DHA-1}$ *dfrA23 qnrB4 sul1 sul2 tet*(A)
FDA00008841	IncC IncI1-Iy	*aph(3'')-Ib aph(6)-Id floR sul2 tet(A) aadA1 bla*$_{TEM-1B}$ *sul3*
FDA00013727	IncC IncI2	*aadA2 aph(3'')-Ib aph(6)-Id bla*$_{DHA-1}$ *dfrA23 floR lnu*(F) *qnrB4 sul1 sul2 tet*(A) *mcr-1.1*
FDA00013728	IncC IncI2	*aadA2 aph(3'')-Ib aph(6)-Id bla*$_{DHA-1}$ *dfrA23 floR lnu*(F) *qnrB4 sul1 sul2 tet*(A) *mcr-1.1*
FDA00013729	IncC IncI2	*aadA2 aph(3'')-Ib aph(6)-Id bla*$_{DHA-1}$ *dfrA23 floR lnu*(F) *qnrB4 sul1 sul2 tet*(A) *mcr-1.1*
FDA00013836	IncC	*aadA2 aph(3'')-Ib aph(6)-Id bla*$_{DHA-1}$ *dfrA23 floR lnu*(F) *qnrB4 sul1 sul2 tet*(A)
PDT000424546.1	IncC IncHI2 IncHI2A	*aadA2 aph(3'')-Ib aph(6)-Id bla*$_{DHA-1}$ *dfrA23 floR lnu*(F) *qnrB4 sul1 sul2 tet*(A) *aadA1 bla*$_{TEM-1B}$ *dfrA1 dfrA12 mph*(A) *oqxAB qnrA6*
PDT000424547.1	IncC IncHI2 IncHI2A	*aadA2 aph(3'')-Ib aph(6)-Id bla*$_{DHA-1}$ *dfrA23 floR lnu*(F) *qnrB4 sul1 sul2 tet*(A) *aadA1 bla*$_{TEM-1B}$ *dfrA1 dfrA12 mph*(A) *oqxAB qnrA6*
PNUSAS010879	IncC	*aadA2 aph(3'')-Ib aph(6)-Id bla*$_{DHA-1}$ *dfrA23 floR lnu*(F) *qnrB4 sul1 sul2 tet*(A)
PNUSAS011492	IncC ColE1	*aadA2 aph(3'')-Ib aph(6)-Id bla*$_{DHA-1}$ *dfrA23 floR lnu*(F) *qnrB4 sul1 sul2 tet*(A)
PNUSAS011751	IncC	*aadA2 aph(3'')-Ib aph(6)-Id bla*$_{DHA-1}$ *dfrA23 floR lnu*(F) *qnrB4 sul1 sul2 tet*(A)
PNUSAS038936	None	*aadA2 aph(3'')-Ib aph(6)-Id bla*$_{DHA-1}$ *dfrA23 floR lnu*(F) *qnrB4 sul1 sul2 tet*(A)
PNUSAS051057	IncC IncI1-Iy	*aadA2 aph(3'')-Ib aph(6)-Id bla*$_{DHA-1}$ *dfrA23 floR lnu*(F) *qnrB4 sul1 sul2 tet*(A)
PNUSAS051059	IncC IncI1-Iy	*aadA2 aph(3'')-Ib aph(6)-Id bla*$_{DHA-1}$ *dfrA23 floR lnu*(F) *qnrB4 sul1 sul2 tet*(A)
PNUSAS068759	IncC	*aadA2 aph(3'')-Ib aph(6)-Id bla*$_{DHA-1}$ *dfrA23 floR lnu*(F) *qnrB4 sul1 sul2 tet*(A)
R15.0600	IncC	*aadA2 aph(3'')-Ib aph(6)-Id bla*$_{DHA-1}$ *dfrA23 floR lnu*(F) *qnrB4 sul1 sul2 tet*(A)
R15.0913	IncC	*aadA2 aph(3'')-Ib aph(6)-Id bla*$_{DHA-1}$ *dfrA23 floR lnu*(F) *qnrB4 sul1 sul2 tet*(A)
R15.2697	IncC	*aadA2 aph(3'')-Ib aph(6)-Id bla*$_{DHA-1}$ *dfrA23 floR lnu*(F) *qnrB4 sul1 sul2 tet*(A)
R16.0676	IncC	*aadA2 aph(3'')-Ib aph(6)-Id bla*$_{DHA-1}$ *dfrA23 floR lnu*(F) *qnrB4 sul1 sul2 tet*(A)
R16.0696	IncC IncI1-Iy	*aadA2 aph(3'')-Ib aph(6)-Id bla*$_{DHA-1}$ *dfrA23 floR lnu*(F) *qnrB4 sul1 sul2 tet*(A)
R16.1070	IncC	*aadA2 aph(3'')-Ib aph(6)-Id bla*$_{DHA-1}$ *dfrA23 floR lnu*(F) *qnrB4 sul1 sul2 tet*(A)
R16.1486	IncC	*aadA2 aph(3'')-Ib aph(6)-Id bla*$_{DHA-1}$ *dfrA23 floR lnu*(F) *qnrB4 sul1 sul2 tet*(A)
R16.2802	IncC	*aadA2 aph(3'')-Ib aph(6)-Id bla*$_{DHA-1}$ *dfrA23 floR lnu*(F) *qnrB4 sul1 sul2 tet*(A)

Strain	Plasmid replicons	Resistance determinants
R16.2885	IncC	*aadA2 aph(3'')-Ib aph(6)-Id bla*$_{DHA-1}$ *dfrA23 floR lnu*(F) *qnrB4 sul1 sul2 tet*(A)
R16.3355	IncC IncI1-Iy	*aadA2 aph(3'')-Ib aph(6)-Id bla*$_{DHA-1}$ *dfrA23 floR lnu*(F) *qnrB4 sul1 sul2 tet*(A) *bla*$_{TEM-1B}$
R16.4304	IncC	*aadA2 aph(3'')-Ib aph(6)-Id bla*$_{DHA-1}$ *dfrA23 floR lnu*(F) *qnrB4 sul1 sul2 tet*(A)
R16.4880	IncC IncX4	*aadA2 aph(3'')-Ib aph(6)-Id bla*$_{DHA-1}$ *dfrA23 floR lnu*(F) *qnrB4 sul1 sul2 tet*(A)
R17.0132	IncC IncI2 IncX4	*aadA2 aph(3'')-Ib aph(6)-Id bla*$_{DHA-1}$ *dfrA23 floR lnu*(F) *qnrB4 sul1 sul2 tet*(A) *mcr-1.1*
R17.3086	IncC	*aadA2 aph(3'')-Ib aph(6)-Id bla*$_{DHA-1}$ *dfrA23 floR lnu*(F) *qnrB4 sul1 sul2 tet*(A)
R17.3110	IncC	*aadA2 aph(3'')-Ib aph(6)-Id* bla$_{DHA-1}$ *dfrA23 floR lnu*(F) *qnrB4 sul1 sul2 tet*(A)
R17.3140	IncC	*aadA2 aph(3'')-Ib aph(6)-Id bla*$_{DHA-1}$ *dfrA23 floR lnu*(F) *qnrB4 sul1 sul2 tet*(A)
R17.3154	IncC	*aadA2 aph(3'')-Ib aph(6)-Id bla*$_{DHA-1}$ *dfrA23 floR lnu*(F) *qnrB4 sul1 sul2 tet*(A)
R17.3160	IncC IncFII(p96A)	*aadA2 aph(3'')-Ib aph(6)-Id bla*$_{DHA-1}$ *dfrA23 floR lnu*(F) *qnrB4 sul1 sul2 tet*(A)
R17.3161	IncC IncFII(p96A)	*aadA2 aph(3'')-Ib aph(6)-Id bla*$_{DHA-1}$ *dfrA23 floR lnu*(F) *qnrB4 sul1 sul2 tet*(A)
R17.3203	IncC	*aadA2 aph(3'')-Ib aph(6)-Id bla*$_{DHA-1}$ *dfrA23 floR lnu*(F) *qnrB4 sul1 sul2 tet*(A)
R17.3211	IncC	*aadA2 aph(3'')-Ib aph(6)-Id bla*$_{DHA-1}$ *dfrA23 floR lnu*(F) *qnrB4 sul1 sul2 tet*(A)
R17.4426	IncC	*aadA2 aph(3'')-Ib aph(6)-Id bla*$_{DHA-1}$ *dfrA23 floR lnu*(F) *qnrB4 sul1 sul2 tet*(A)
R17.4643	IncC	*aadA2 aph(3'')-Ib aph(6)-Id bla*$_{DHA-1}$ *dfrA23 floR lnu*(F) *qnrB4 sul1 sul2 tet*(A)
R17.5171	IncC	*aadA2 aph(3'')-Ib aph(6)-Id bla*$_{DHA-1}$ *dfrA23 floR lnu*(F) *qnrB4 sul1 sul2 tet*(A)
R18.1458	IncC	*aadA2 aph(3'')-Ib aph(6)-Id bla*$_{DHA-1}$ *dfrA23 floR lnu*(F) *qnrB4 sul1 sul2 tet*(A)

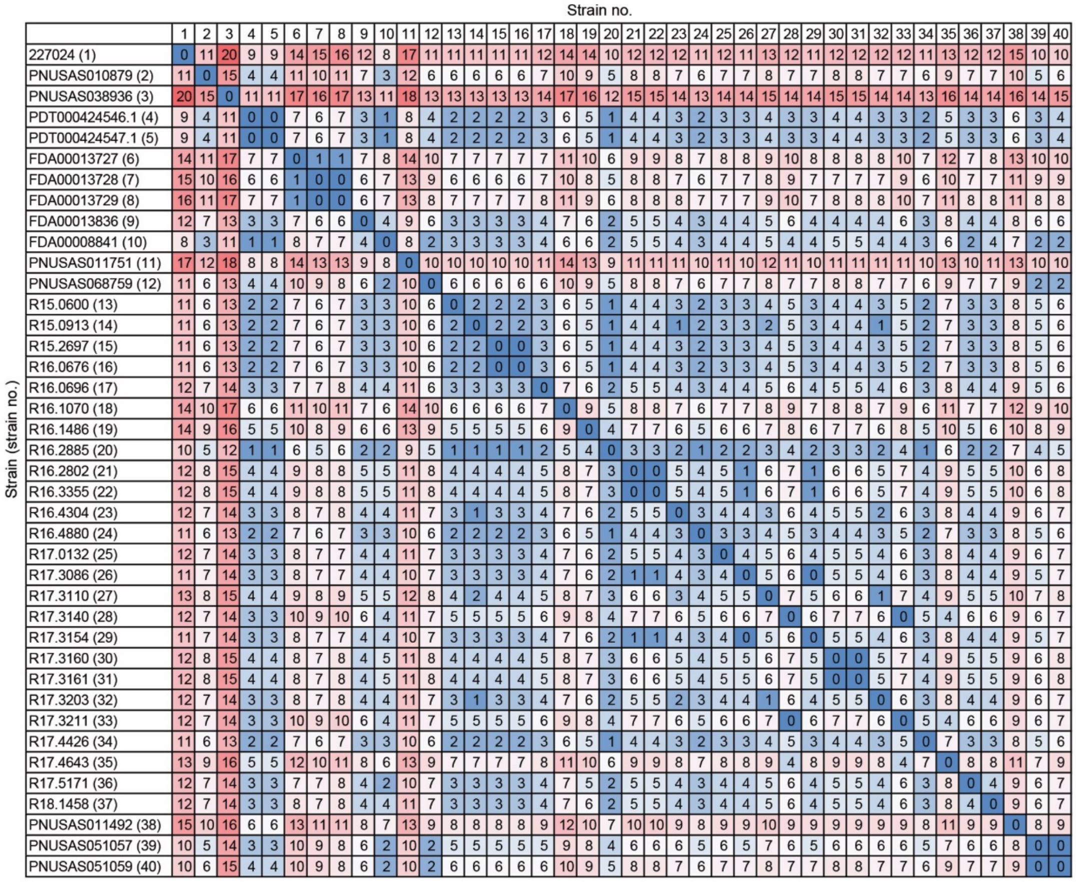

	1	2	3	4	5	6	7	8	9	10	11	12	13	14	15	16	17	18	19	20	21	22	23	24	25	26	27	28	29	30	31	32	33	34	35	36	37	38	39	40
227024 (1)	0	11	20	9	9	14	15	16	12	8	17	11	11	11	11	11	12	14	14	10	12	12	12	11	12	11	13	12	11	12	12	12	12	11	13	12	12	15	10	10
PNUSAS010879 (2)	11	0	15	4	4	11	10	11	7	3	12	6	6	6	6	6	7	10	9	5	8	8	7	6	7	7	8	7	7	8	8	7	7	6	9	7	7	10	5	6
PNUSAS038936 (3)	20	15	0	11	11	17	16	17	13	11	18	13	13	13	13	13	14	17	16	12	15	15	14	13	14	14	15	14	14	15	15	14	14	13	16	14	14	16	14	15
PDT000424546.1 (4)	9	4	11	0	0	7	6	7	3	1	8	4	2	2	2	2	3	6	5	1	4	4	3	2	3	3	4	3	3	4	4	3	3	2	5	3	3	6	3	4
PDT000424547.1 (5)	9	4	11	0	0	7	6	7	3	1	8	4	2	2	2	2	3	6	5	1	4	4	3	2	3	3	4	3	3	4	4	3	3	2	5	3	3	6	3	4
FDA00013727 (6)	14	11	17	7	7	0	1	1	7	8	14	10	7	7	7	7	7	11	10	6	9	9	8	7	8	8	9	10	8	8	8	8	10	7	12	7	8	13	10	10
FDA00013728 (7)	15	10	16	6	6	1	0	0	6	7	13	9	6	6	6	6	7	10	8	5	8	8	7	6	7	7	8	9	7	7	7	7	9	6	10	7	7	11	9	9
FDA00013729 (8)	16	11	17	7	7	1	0	0	6	7	13	8	7	7	7	7	8	11	9	6	8	8	8	7	7	7	9	10	7	8	8	8	10	7	11	8	8	11	8	8
FDA00013836 (9)	12	7	13	3	3	7	6	6	0	4	9	6	3	3	3	3	4	7	6	2	5	5	4	3	4	4	5	6	4	4	4	4	6	3	8	4	4	8	6	6
FDA00008841 (10)	8	3	11	1	1	8	7	7	4	0	8	2	3	3	3	3	4	6	6	2	5	5	4	3	4	4	5	4	4	5	5	4	4	3	6	2	4	7	2	2
PNUSAS011751 (11)	17	12	18	8	8	14	13	13	9	8	0	10	10	10	10	10	11	14	13	9	11	11	11	10	11	10	12	11	10	11	11	11	11	10	13	10	11	13	10	10
PNUSAS068759 (12)	11	6	13	4	4	10	9	8	6	2	10	0	6	6	6	6	6	10	9	5	8	8	7	6	7	7	8	7	7	8	8	7	7	6	9	7	7	9	2	2
R15.0600 (13)	11	6	13	2	2	7	6	7	3	3	10	6	0	2	2	2	3	6	5	1	4	4	3	2	3	3	4	5	3	4	4	3	5	2	7	3	3	8	5	6
R15.0913 (14)	11	6	13	2	2	7	6	7	3	3	10	6	2	0	2	2	3	6	5	1	4	4	1	2	3	3	2	5	3	4	4	1	5	2	7	3	3	8	5	6
R15.2697 (15)	11	6	13	2	2	7	6	7	3	3	10	6	2	2	0	0	3	6	5	1	4	4	3	2	3	3	4	5	3	4	4	3	5	2	7	3	3	8	5	6
R16.0676 (16)	11	6	13	2	2	7	6	7	3	3	10	6	2	2	0	0	3	6	5	1	4	4	3	2	3	3	4	5	3	4	4	3	5	2	7	3	3	8	5	6
R16.0696 (17)	12	7	14	3	3	7	7	8	4	4	11	6	3	3	3	3	0	7	6	2	5	5	4	3	4	4	5	6	4	5	5	4	6	3	8	4	4	9	5	6
R16.1070 (18)	14	10	17	6	6	11	10	11	7	6	14	10	6	6	6	6	7	0	9	5	8	8	7	6	7	7	8	9	7	8	8	7	9	6	11	7	7	12	9	10
R16.1486 (19)	14	9	16	5	5	10	8	9	6	6	13	9	5	5	5	5	6	9	0	4	7	7	6	5	6	6	7	8	6	7	7	6	8	5	10	5	6	10	8	9
R16.2885 (20)	10	5	12	1	1	6	5	6	2	2	9	5	1	1	1	1	2	5	4	0	3	3	2	1	2	2	3	4	2	3	3	2	4	1	6	2	2	7	4	5
R16.2802 (21)	12	8	15	4	4	9	8	8	5	5	11	8	4	4	4	4	5	8	7	3	0	0	5	4	5	1	6	7	1	6	6	5	7	4	9	5	5	10	6	8
R16.3355 (22)	12	8	15	4	4	9	8	8	5	5	11	8	4	4	4	4	5	8	7	3	0	0	5	4	5	1	6	7	1	6	6	5	7	4	9	5	5	10	6	8
R16.4304 (23)	12	7	14	3	3	8	7	8	4	4	11	7	3	1	3	3	4	7	6	2	5	5	0	3	4	4	3	6	4	5	5	2	6	3	8	4	4	9	6	7
R16.4880 (24)	11	6	13	2	2	7	6	7	3	3	10	6	2	2	2	2	3	6	5	1	4	4	3	0	3	3	4	5	3	4	4	3	5	2	7	3	3	8	5	6
R17.0132 (25)	12	7	14	3	3	8	7	7	4	4	11	7	3	3	3	3	4	7	6	2	5	5	4	3	0	4	5	6	4	5	5	4	6	3	8	4	4	9	6	7
R17.3086 (26)	11	7	14	3	3	8	7	7	4	4	10	7	3	3	3	3	4	7	6	2	1	1	4	3	4	0	5	6	0	5	5	4	6	3	8	4	4	9	5	7
R17.3110 (27)	13	8	15	4	4	9	8	9	5	5	12	8	4	2	4	4	5	8	7	3	6	6	3	4	5	5	0	7	5	6	6	1	7	4	9	5	5	10	7	8
R17.3140 (28)	12	7	14	3	3	10	9	10	6	4	11	7	5	5	5	5	6	9	8	4	7	7	6	5	6	6	7	0	6	7	7	6	0	5	4	6	6	9	6	7
R17.3154 (29)	11	7	14	3	3	8	7	7	4	4	10	7	3	3	3	3	4	7	6	2	1	1	4	3	4	0	5	6	0	5	5	4	6	3	8	4	4	9	5	7
R17.3160 (30)	12	8	15	4	4	8	7	8	4	5	11	8	4	4	4	4	5	8	7	3	6	6	5	4	5	5	6	7	5	0	0	5	7	4	9	5	5	9	6	8
R17.3161 (31)	12	8	15	4	4	8	7	8	4	5	11	8	4	4	4	4	5	8	7	3	6	6	5	4	5	5	6	7	5	0	0	5	7	4	9	5	5	9	6	8
R17.3203 (32)	12	7	14	3	3	8	7	8	4	4	11	7	3	1	3	3	4	7	6	2	5	5	2	3	4	4	1	6	4	5	5	0	6	3	8	4	4	9	6	7
R17.3211 (33)	12	7	14	3	3	10	9	10	6	4	11	7	5	5	5	5	6	9	8	4	7	7	6	5	6	6	7	0	6	7	7	6	0	5	4	6	6	9	6	7
R17.4426 (34)	11	6	13	2	2	7	6	7	3	3	10	6	2	2	2	2	3	6	5	1	4	4	3	2	3	3	4	5	3	4	4	3	5	0	7	3	3	8	5	6
R17.4643 (35)	13	9	16	5	5	12	10	11	8	6	13	9	7	7	7	7	8	11	10	6	9	9	8	7	8	8	9	4	8	9	9	8	4	7	0	8	8	11	7	9
R17.5171 (36)	12	7	14	3	3	7	7	8	4	2	10	7	3	3	3	3	4	7	5	2	5	5	4	3	4	4	5	6	4	5	5	4	6	3	8	0	4	9	6	7
R18.1458 (37)	12	7	14	3	3	8	7	8	4	4	11	7	3	3	3	3	4	7	6	2	5	5	4	3	4	4	5	6	4	5	5	4	6	3	8	4	0	9	6	7
PNUSAS011492 (38)	15	10	16	6	6	13	11	11	8	7	13	9	8	8	8	8	9	12	10	7	10	10	9	8	9	9	10	9	9	9	9	9	9	8	11	9	9	0	8	9
PNUSAS051057 (39)	10	5	14	3	3	10	9	8	6	2	10	2	5	5	5	5	5	9	8	4	6	6	6	5	6	5	7	6	5	6	6	6	6	5	7	6	6	8	0	0
PNUSAS051059 (40)	10	6	15	4	4	10	9	8	6	2	10	2	6	6	6	6	6	10	9	5	8	8	7	6	7	7	8	7	7	8	8	7	7	6	9	7	7	9	0	0

Appendix Figure. Core genome multilocus sequence typing (cgMLST) pairwise matrix of allele differences for 40 *Salmonella* Anatum isolates. Cell values indicate the number of allele differences between isolates. The matrix was constructed by using BioNumerics version 7.6 (Applied Maths, http://www.applied-maths.com). Based on the number of allele differences, cells are shaded from dark blue (0 allele differences) to red (20 allele differences). National Center for Biotechnology Information strain or isolate numbers are shown.
